# *Lactobacillus gasseri* APC 678 Reduces Shedding of the Pathogen *Clostridium difficile* in a Murine Model

**DOI:** 10.3389/fmicb.2019.00273

**Published:** 2019-02-20

**Authors:** Lisa Quigley, Mairéad Coakley, Debebe Alemayehu, Mary C. Rea, Patrick G. Casey, Órla O’Sullivan, Eileen Murphy, Barry Kiely, Paul D. Cotter, Colin Hill, R. Paul Ross

**Affiliations:** ^1^Teagasc Food Research Centre, Moorepark, Fermoy, Co. Cork, Ireland; ^2^APC Microbiome Ireland, University College Cork, Cork, Ireland; ^3^School of Microbiology, University College Cork, Cork, Ireland; ^4^Alimentary Health Ltd., Cork, Ireland

**Keywords:** *Lactobacillus gasseri*, *Clostridium difficile*, *C. difficile* infection (CDI), murine model, live therapeutic agent

## Abstract

*Clostridium difficile* is a common cause of health-care acquired diarrhea, resulting in a spectrum of disease from mild diarrhea to life-threatening illness. Sixty *Lactobacillus* strains were screened for anti-*C. difficile* activity using a co-culture method. Based on their ability to inhibit *C. difficile*, *L. gasseri* APC 678 and *L. rhamnosus* DPC 6111 were selected for study in a murine model of *C. difficile* infection. *L. gasseri* ATCC 33323, was included as a control. It was established that, relative to control mice not fed *Lactobacillus*, feeding with *L. gasseri* APC 678 resulted in a significant reduction by day 7 (8-fold, *p* = 0.017) of viable *C. difficile* VPI 10463 in the feces of mice. In contrast, neither *L. rhamnosus* DPC 6111 nor *L. gasseri* ATCC 33323 significantly reduced fecal *C. difficile* shedding. Sequencing of the cecal microbiota showed that in mice fed *L. gasseri* APC 678 there was a significant increase in bacterial diversity across a number of indices when compared to the control or other *Lactobacillus*-fed groups. There was no significant change in the relative abundance of Firmicutes or Bacteroidetes in the group fed *L. gasseri* APC 678 relative to the control, while the groups fed *L. rhamnosus* DPC 6111 or *L. gasseri* ATCC 33323 showed a significant decrease in the relative abundance of Firmicutes (*p* = 0.002 and *p* = 0.019, respectively) and a significant increase in Bacteroidetes (*p* = 0.002 and *p* = 0.023, respectively). These results highlight the potential of *L. gasseri* APC 678 as a live therapeutic agent to target *C. difficile* infection.

## Introduction

*Clostridium difficile* is a Gram positive, cytotoxin-producing anaerobic intestinal pathogen with an asymptomatic carriage rate of up to 30% in people in long-term care facilities (Ziakas et al., 2015). The bacterium has recently been reclassified as *Clostridioides difficile* (Lawson et al., 2016). When the human intestinal microbiota is altered following broad spectrum antibiotic therapy, *C. difficile* may flourish and cause illness, varying from mild diarrhea (usually self-limiting) to pseudomembranous colitis, fulminant colitis, toxic mega-colon and even death (Kachrimanidou and Malisiovas, 2011). The incidences of CDI have rapidly increased since the 1990s, and the mortality rate has also grown markedly (Wiegand et al., 2012). Recent studies indicate that the economic burden of *C. difficile* is mounting as a result of increased incidences of infection in hospitalized patients. Estimates show that the economic health-care costs of CDI are over $4.8 billion per annum in the United States and over €3 billion per annum in Europe (DePestel and Aronoff, 2013).

The ESCMID guidelines for treatment of CDI include antibiotics, toxin-binding resins and polymers, immunotherapy, probiotics and fecal or bacterial intestinal transplantation (Debast et al., 2014). Antibiotic treatment is typically advised, including the use of metronidazole, vancomycin and fidaxomicin (Debast et al., 2014). However, as standard therapies for CDI frequently have limited efficacy, the search for alternative therapies including live therapeutics and bacteriocins such as thuricin CD that may reduce incidences and recurring infections are gaining credence (Rea et al., 2013; Evans and Johnson, 2015; Goldstein et al., 2015). One of the strategies used to modulate the gut microbiota is the dietary administration of live microorganisms (Hill et al., 2014). A meta-analysis of the literature, from 1985 to 2013, found that the use of probiotics significantly prevented CDI and antibiotic-associated diarrhea in children, but that the effect of the probiotics was strain dependent (McFarland and Goh, 2013). The mode of action of live therapeutics is multi-faceted and includes an improvement in epithelial barrier function, immune-modulation, secretion of antimicrobial substances (e.g., bacteriocins and hydrogen peroxide) and bioactive metabolites (e.g., CLA), inhibition of the expression of virulence factors, playing a role in competitive exclusion possibly through colonization resistance or through the production of neurotransmitters such as GABA, which may impact on brain function (Dobson et al., 2012; O’Shea et al., 2012; Sultana et al., 2013; Nebot-Vivinus et al., 2014; Dinan et al., 2015; Fernandez et al., 2015). However, the effect of pure cultures of bacterial strains administered as live therapeutics has been shown in many instances to be strain rather than species dependent indicating that careful strain selection is required (Wall et al., 2012). Lactobacilli are commonly used as probiotics, having health impacts as outlined above, with a range of lactobacilli (including *L. gasseri* and *L. rhamnosus*) on the EFSA list of microorganisms suitable for use in food and feed production (Ricci et al., 2017).

More recently FMT has been used with some success for the treatment of refractory CDI and interest in this area has risen as evidenced by the large increase in publications relating to FMT over the last number of years (Bojanova and Bordenstein, 2016). To date the mechanism of action of FMT to break the cycle of recurrent CDI has remained poorly understood. However, intra-colonic bile acid has been suggested to play a role in both spore germination and inhibition of vegetative cells of *C. difficile* (Weingarden et al., 2016). *Clostridium scindens*, a member of the intestinal microbiota capable of dehydroxylating bile acid, was shown to be associated with resistance to *C. difficile* and enhanced resistance to infection in a murine model of CDI (Buffie et al., 2015).

Here, we demonstrate how screening specifically for anti-*C. difficile* lactobacilli identified a strain from the human GIT with the ability to reduce *C. difficile* shedding in a murine model of CDI.

## Materials and Methods

### Bacterial Strains and Growth Conditions

*Lactobacillus* strains were maintained at -80°C in 40% (v/v) glycerol and routinely cultured anaerobically at 37°C on MRS agar (Difco, Becton Dickinson, Franklin Lakes, NJ, United States) for 48 h or overnight in MRS broth. *C. difficile* strains EM304 (ribotype 027) and VPI 10463 (see Supplementary Table 1 for details) were maintained at -80°C on micro-bank beads (Pro-Lab Diagnostics, Merseyside, United Kingdom) and cultured on Fastidious Anaerobic Agar (Lab M, Heywood, Lancashire, United Kingdom) supplemented with 7% defibrinated horse blood (Cruinn Diagnostics, Dublin, Ireland) at 37°C for 3 days. Fresh cultures were grown overnight at 37°C in RCM (Merck, Darmstadt, Germany), pre-boiled and cooled under anaerobic conditions. The lactobacilli and clostridia were grown in an anaerobic chamber (Don Whitley, West Yorkshire, United Kingdom) under an anoxic atmosphere (10% CO_2_, 10% H_2_, 80% N_2_), unless otherwise stated.

### Screening of *Lactobacillus* Strains for Anti-bacterial Activity Against *Clostridium difficile*

#### Agar Diffusion Assay

One thousand five hundred *Lactobacillus* isolates of food, human and animal origin were assessed for anti-bacterial activity against *C. difficile* EM304 using agar diffusion assays. *Lactobacillus* strains were grown overnight on MRS agar at 37°C anaerobically. *C. difficile* cells were grown to mid log phase in RCM and harvested at an optical density (OD_600__nm_) of 0.8 and washed twice in preconditioned (pre-boiled and cooled under anaerobic conditions) MRD (Oxoid Ltd, Basingstoke, England) and re-suspended in MRD before use. One hundred microliters of the washed *C. difficile* cells were mixed with 5 ml BHI agar (0.7% agar, BHI, Oxoid) and poured onto BHI agar. *Lactobacillus* colonies were stabbed from the MRS agar into the *C. difficile* lawn with an inoculating needle. Plates were incubated anaerobically for 24 h and assessed for zones of inhibition.

In addition *L. gasseri* APC 678 was assessed for bacteriocin activity against a range of target organisms, as previously described (Rea et al., 2010). The full list of target strains, together with their growth conditions are outlined in Supplementary Table 2.

#### Co-culture Broth

Sixty *Lactobacillus* strains (Table 1) of human and animal origin were selected for screening using a co-culture method, with *C. difficile* EM304 (ribotype 027) (Rea et al., 2012) as the target strain. A MGM was developed to reflect the limited availability of simple sugars in the human gut and was buffered to prevent pH drop during incubation to enable co-culturing of *Lactobacillus* and *Clostridium* strains. The growth medium composition (per liter) was: meat extract, 2 g; peptone, 2 g; yeast extract, 1 g; NaCl, 5 g; sodium acetate, 0.5 g; L-cysteine hydrochloride, 0.5 g; glucose, 0.1 g; NaH_2_PO_4_.H_2_0, 3.7 g; Na_2_HPO_4_.7H_2_O, 6.2 g; pH 6.8 (±0.2). Following overnight growth, 1 ml of *C. difficile* EM304 and each *Lactobacillus* strain were centrifuged at 14,000 × *g*. Pelleted cells were washed once in PBS under anaerobic conditions and re-suspended in fresh PBS. The MGM was inoculated with a test strain of *Lactobacillus* and *C. difficile* EM304 at ∼10^6^ CFU ml^-1^. The cultures were then incubated at 37°C for 24 h. Survival of *C. difficile* was determined by plating onto Brazier’s CCEY (Lab M) and *Lactobacillus* counts were determined by plating onto MRS agar (Difco). Agar plates were incubated anaerobically at 37°C for 2–3 days and the anti-bacterial activity of the lactobacilli was determined as a reduction in *C. difficile* counts compared to the control in the absence of *Lactobacillus*. *Clostridium difficile* VPI 10463, used in the murine model, was also assessed in co-culture with the three lactobacilli used in the study (*L. gasseri* APC 678, *L. rhamnosus* DPC 6111 and *L. gasseri* ATCC 33323).

**Table 1:** Lactobacilli screened for anti-*Clostridium difficile* activity.

*Lactobacillus* spp.	No. of strains tested	Source
*L. gasseri*	7	Human intestine (2), human feces (5)
*L. salivarius*	6	Pig intestine (2), infant feces (3), human feces (1)
*L. plantarum*	5	Bovine teat rinse, cow feces, silage, hand wash, milking yard water
*L. brevis*	4	Human feces (2), cow feces (1), silage (1)
*L. casei/paracasei*	4	Human feces
*L. paracasei*	4	Human intestine (2), human feces (1), yoghurt (1)
*L. rhamnosus*	4	Human intestine (1), human feces (3)
*L. mucosae*	3	Cow feces
*L. acidophilus*	2	Human feces
*L. casei*	2	Fermented milk
*L. johnsonii*	2	Pig intestine, commercial isolate
*L. parabuchneri/kefiri*	2	Milk, cow feces
*L. reuteri*	2	Pig intestine, infant feces
*L. rhamnosus/casei*	2	Human feces
*L. ruminis*	2	Human feces
*L. murinus*	1	Pig cecum
*Lactobacillus* spp.	8	Human feces

#### Survival of Lactobacilli During *in vitro* Gastrointestinal Transit

The ability of the bacterial strains to survive in a simulated gastric environment was assessed. Briefly, MRS broth was inoculated at 1% with the *Lactobacillus* strains and incubated anaerobically at 37°C for 16 h. One milliliter of cells was centrifuged at 14,000 × *g*, washed in PBS and re-centrifuged. The cells were then re-suspended in PBS or 10% RSM. A suspension of 10^8^ CFU ml^-1^ *Lactobacillus* was suspended in artificial gastric juice with the following composition (per liter): NaCl, 125 mmol; KCl, 7 mmol; NaHCO_3_, 45 mmol and pepsin, 3 g. The final pH was adjusted with HCl to pH 2 or pH 3 or with NaOH to pH 7. The bacterial suspensions were incubated at 37°C with agitation (200 rpm). Viable cells were enumerated at 0, 90 and 180 min.

Following 180 min suspension in simulated gastric juice, the cells were suspended in simulated intestinal fluid, which was prepared with 0.10% (w/v) pancreatin (Sigma Aldrich, Ireland) and 0.15% oxgall bile salts (Difco) in water, adjusted to pH 8.0 with NaOH for a further 180 min. The suspensions were incubated at 37°C and samples taken to assess viability on MRS agar at 90 and 180 min. Plates were incubated at 37°C for 48 h. Survival was expressed as log reduction from 0 h.

### *In vivo* Assessment of *Lactobacillus* Strains in *C. difficile* Murine Model

#### Ethics Statement

All procedures involving animals were approved by the University College Cork Animal Experimentation Ethics Committee (#2011/17) and by the HPRA. Animals were sourced from Harlan Laboratories UK, Bicester, Oxfordshire, United Kingdom. At the conclusion of the experiment animals were euthanized by cervical dislocation.

#### Mouse Model

For the *C. difficile* model, 40 female C57BL/6 (7 weeks old) mice were obtained from Harlan Laboratories UK. All mice used in the experiment were housed in groups of five animals per cage under the same conditions. Food, water, bedding and cages were autoclaved before use.

#### Antibiotic Administration

All mice were made susceptible to CDI by altering the gut microbiota using a previously described protocol (Chen et al., 2008). Briefly, an antibiotic mixture comprising of kanamycin (0.4 mg mL^-1^), gentamicin (0.035 mg mL^-1^), colistin (850 U mL^-1^), metronidazole (0.215 mg L^-1^) and vancomycin (0.045 mg mL^-1^) was prepared in water (all antibiotics were purchased from Sigma). This corresponded to an approximate daily dose for each antibiotic of: kanamycin, 40 mg kg^-1^; gentamicin, 3.5 mg kg^-1^; colistin, 4.2 mg kg^-1^; metronidazole, 21.5 mg kg^-1^ and vancomycin, 4.5 mg kg^-1^. The concentrations of antibiotics in the water were calculated based on the average weight of the animals and expected daily water consumption of the mice. All mice received the antibiotic cocktail in water for 3 days, followed by 2 days of water without antibiotics. All mice received a single dose of clindamycin 10 mg kg^-1^ intraperitoneally 1 day before *C. difficile* challenge.

#### Preparation of Bacterial Cultures

Adhering to strict anaerobic conditions, *C. difficile* VPI 10463 was grown overnight in RCM. Bacterial cells were collected by centrifugation at 4,050 × *g* for 5 min, washed once in preconditioned PBS and re-suspended in PBS to achieve a preparation of 5 × 10^5^ CFU per mouse. *Lactobacillus* strains for each group; *L. gasseri* APC 678, *L. rhamnosus* DPC 6111 or *L. gasseri* ATCC 33323 (see Supplementary Table 1 for details) were prepared by growing the strains overnight in MRS broth under anaerobic conditions. Cells were collected by centrifugation at 4,050 × *g* for 5 min, washed once in preconditioned saline solution and re-suspended in 10% (w/v) RSM to achieve 1 × 10^9^ CFU ml^-1^. The control group was fed 10% RSM only. At the start of the experiment all mice (10/group) received an individual inoculum of *C. difficile* (5 × 10^5^ CFU/mouse). Five hours later, 100 μl of the appropriate probiotic (equivalent to 1 × 10^8^ CFU) or RSM (control group) was administered by oral gavage, and then daily for 7 days.

#### Sample Collection and *C. difficile* Counts

Prior to commencement of the trial and before antibiotic treatment, fecal samples were collected from all animals and plated on CCEY agar to confirm that the mice were *C. difficile*-free. Subsequently, fecal pellets were collected at 24 h, 4, and 7 days post-infection with *C. difficile* and stored anaerobically before being assessed for viable *C. difficile* (CFU g^-1^ feces). At the end of the trial the mice were sacrificed and total numbers of *C. difficile* per colon were counted (CFU colon^-1^). *C. difficile* survival was determined by culturing anaerobically at 37°C on CCEY agar for 48 h. The putative *C. difficile* colonies were confirmed using a *C. difficile* test kit (Oxoid). Following euthanasia, cecal contents were collected for compositional sequencing from each individual mouse, snap frozen and stored at -80°C until required.

### Microbial DNA Extraction, 16S rRNA Amplification and Illumina MiSeq Sequencing

Total metagenomic DNA was extracted for each mouse cecum (sacrificed 7 days post-infection with *C. difficile*), following thawing at 4°C, with the QIAamp DNA Stool Mini Kit (Qiagen, Hilden, Germany) with an additional bead beating step (Murphy et al., 2010). DNA was quantified using the Nanodrop 1000 spectrophotometer (Thermo Fisher Scientific, Waltham, MA, United States). Initially the template DNA was amplified using primers specific to the V3-V4 region of the 16S rRNA gene which also allowed for the Illumina overhang adaptor, where the forward (5′TCGTCGGCAGCGTCAGATGTGTATAAGAGACAGCCTA CGGGNGGCWGCAG) and reverse primers (5′GTCTCGTGGG CTCGGAGATGTGTATAAGAGACAGGACTACHVGGGTATC TAATCC) were used. Each PCR reaction contained 2.5 μl DNA template (5 ng), 5 μl forward primer (1 μM), 5 μl reverse primer (1 μM) (Sigma) and 12.5 μl Kapa HiFi Hotstart Readymix (2X) (Anachem). The template DNA was amplified under the following PCR conditions: 95°C for 3 min (initialization); 95°C for 30 s (denaturation), 55°C for 30 s (annealing), and 72°C for 30 s (elongation) (for a total of 25 cycles); followed by a final elongation step at 72°C for 5 min. PCR products were visualized using gel electrophoresis (1X TAE buffer, 1.5% agarose gel, 100 V). Successful amplicons were cleaned using the AMPure XP purification system (Labplan, Kildare, Ireland). A second PCR reaction was completed using the previously amplified and purified DNA as the template. Two indexing primers (Nextera XT indexing primers, Illumina, Sweden) were used per sample to allow all samples to be pooled, sequenced on one flow cell and subsequently identified bioinformatically. Each reaction contained 25 μl Kapa HiFi HotStart ReadyMix (2X), 5 μl template DNA, 5 μl index primer 1 (N7XX), 5 μl index primer 2 (S5XX) and 10 μl PCR grade water. PCR conditions were the same as previously described with the samples undergoing 8 cycles instead of 25 cycles. Samples were quantified using the Qubit 2.0 fluorometer (Invitrogen) in conjunction with the broad range DNA quantification assay kit (Thermo Fisher Scientific). All samples were pooled to an eqimolar concentration. The quality of the pool was determined by running on the Agilent Bioanalyser prior to sequencing. The sample pool was then denatured with 0.2 M NaOH, diluted to 4 pM and combined with 10% (v/v) denatured 4 pM PhiX. Samples were sequenced on the Illumina MiSeq (Teagasc Sequencing Centre, Moorepark, Fermoy, Co. Cork, Ireland) using a 2300 cycle V3 kit, following protocols outlined by Illumina.

#### Bioinformatic Analysis

Raw Illumina 300 base pair paired-end sequence reads were merged using Flash (Magoc and Salzberg, 2011) and quality checked using the split libraries script from the QIIME package (Caporaso et al., 2010). Reads were then clustered into OTUs and chimeras removed with the 64-bit version of USEARCH (Edgar, 2010). Subsequently OTUs were aligned and a phylogenetic tree generated within QIIME. Taxonomical assignments were reached using the SILVA 16S specific database (version 111) (Quast et al., 2013). Alpha and beta diversity analysis was also implemented within QIIME. PCoA plots were visualized using R (version 3.2.2).

### Statistical Analysis

Non-parametric statistical analyses (Mann Whitney) were applied on MiniTab (Version 15) and SPSS (PASW Statistics version 18) statistical packages, to assess whether differences in *C. difficile* shedding, microbiota composition and diversity between the control and probiotic-fed groups were significant. Statistical significance was accepted at *p* < 0.05, adjusted for ties, where the null hypothesis was rejected.

### Accession Number(s)

Sequence data have been deposited to the ENA under project accession number PRJEB30242.

## Results

### Screening for Lactobacilli With Anti-bacterial Activity Against *Clostridium difficile*

**Figure 1:**
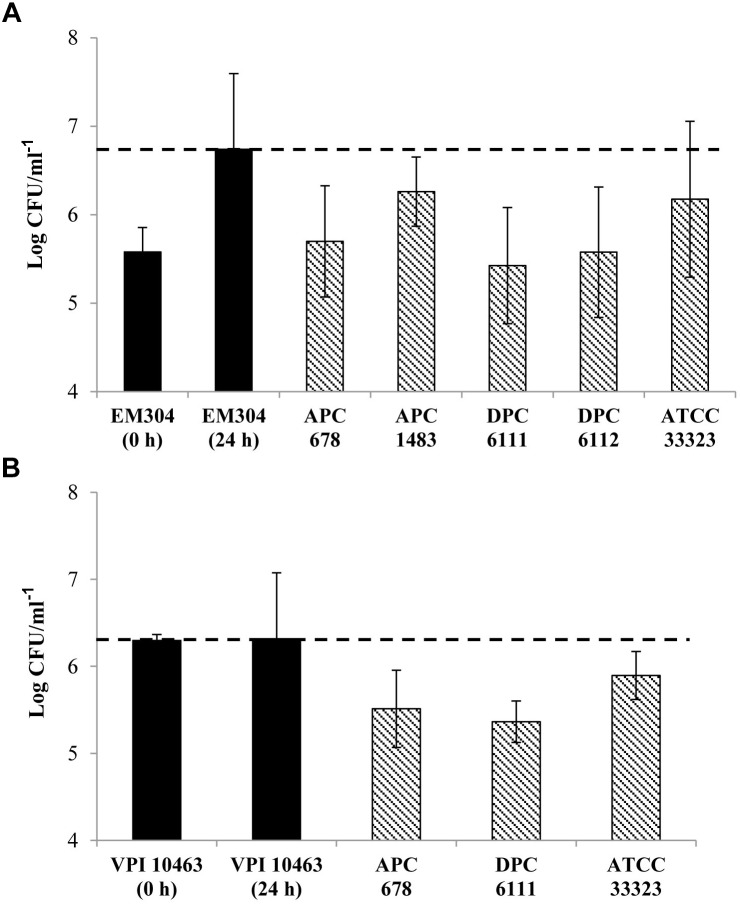
Effect of probiotic lactobacilli on the survival of *Clostridium difficile* in co-culture. Black bars (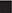) show the cell numbers of *C. difficile* at 0 and 24 h in the absence of *Lactobacillus* strains. Bars with dashed lines (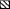) show the cell numbers of **(A)** *C. difficile* EM304 following 24 h co-culture with *L. gasseri* APC 678, *L. paracasei* APC 1483, *L. rhamnosus* DPC 6111, *L. gasseri* DPC 6112 or *L. gasseri* ATCC 33323 and **(B)** *C. difficile* VPI 10463 following 24 h co-culture with *L. gasseri* APC 678, *L. rhamnosus* DPC 6111 or *L. gasseri* ATCC 33323. The horizontal dashed line (---) indicates the final count of *C. difficile* at 24 h in the absence of the *Lactobacillus* strains. The average and standard error of the mean (SEM) of three independent repetitions are represented.

Initial screening of hundreds of *Lactobacillus* isolates for the production of antibacterial compounds against *C. difficile* using an antagonistic agar assay failed to reveal zones of inhibition. Therefore, a low nutrient medium (MGM) was developed which more closely represented the low concentration of simple carbohydrates in the human colon, enabling both *Lactobacillus* and *C. difficile* strains to survive in co-culture over a 24 h period. Due to the buffering capacity of the medium, the pH was maintained at near neutral (∼pH 6.5) following incubation for 24 h, eliminating concerns relating to the reduction of *C. difficile* merely as a result of acid production. Since most of the lactobacilli assessed in the antagonistic agar assay were of dairy origin, *Lactobacillus* strains (*n* = 60) of human, animal, feed and environmental origin were then selected and screened for inhibitory activity against *C. difficile* (Table 1). The co-culture assay showed that 4 of the 60 lactobacilli tested (*L. gasseri* APC 678, *L. paracasei* APC 1483, *L. rhamnosus* DPC 6111 and *L. gasseri* DPC 6112) had the ability to reduce the survival of *C. difficile* EM304 *in vitro* (Figure 1A). The co-culture of *C. difficile* VPI 10463 with each of the three lactobacilli screened in the mouse study (*L. gasseri* APC 678, *L. rhamnosus* DPC 6111 and *L. gasseri* ATCC 33323) also negatively impacted *C. difficile* survival (Figure 1B).

### Simulated Gastrointestinal Fluid Demonstrates Tolerance of Lactobacilli to Digestion

Among the important probiotic traits required for *Lactobacillus* strains intended for use in the GIT is the ability to survive the acidic conditions of the stomach and the presence of bile in the upper small intestine. Here, we demonstrate the survival of *L. gasseri* APC 678 and *L. rhamnosus* DPC 6111 in a simulated GIT environment (Table 2). Both strains, when suspended in PBS before being added to simulated gastric juice at pH 3 were found to be stable. However, *L. gasseri* APC 678 was the more stable of the two at pH 2, showing a 1.5 log reduction in total viable counts compared to 3.5 log reduction for *L. rhamnosus* DPC 6111. Viable counts indicated that neither strain survived the subsequent 3 h incubation in simulated ileal juice. However, if the strains were suspended in 10% RSM prior to treatment with gastric/ileal juice, their survival was markedly improved both in simulated gastric juice at both pH 2 and 3 and also after a further 3 h incubation in ileal juice (Table 2). In 10% RSM *L. rhamnosus* DPC 6111 was more sensitive to the overall conditions of the stomach and ileum than *L. gasseri* APC 678, showing a 4.4 log reduction at the end of the incubation period compared with a reduction of 2.8 log for *L. gasseri* APC 678.

**Table 2:** Survival of lactobacilli during simulated gastrointestinal tract transit.

		Log reduction (CFU ml^-1^) after 3 h in simulated gastric juice		Log reduction (CFU ml^-1^) after 3 h in simulated ileal juice^∗^	
Suspension medium	pH	*L. gasseri* APC 678	*L. rhamnosus* DPC 6111	*L. gasseri* APC 678	*L. rhamnosus* DPC 6111
PBS	7.0	0.16	0.21	0.52	0.43
	3.0	1.96	1.01	2.19	1.78
	2.0	1.52	3.53	8.00	8.00
RSM	7.0	0.84	0.49	0.52	0.13
	3.0	1.92	0.91	1.89	2.47
	2.0	1.91	1.49	2.82	4.39

*The effect of initial suspension medium (PBS or RSM) on the subsequent survival of *L. gasseri* APC 678 and *L. rhamnosus* DPC 6111 in simulated gastric juice, followed by simulated ileal juice. ^∗^Cells were transferred to simulated ileal juice following 3 h incubation in gastric juice and incubated for an additional 3 h*.

### *Lactobacillus* Strains Reduce *C. difficile* Shedding in a Mouse Model

**Figure 2:**
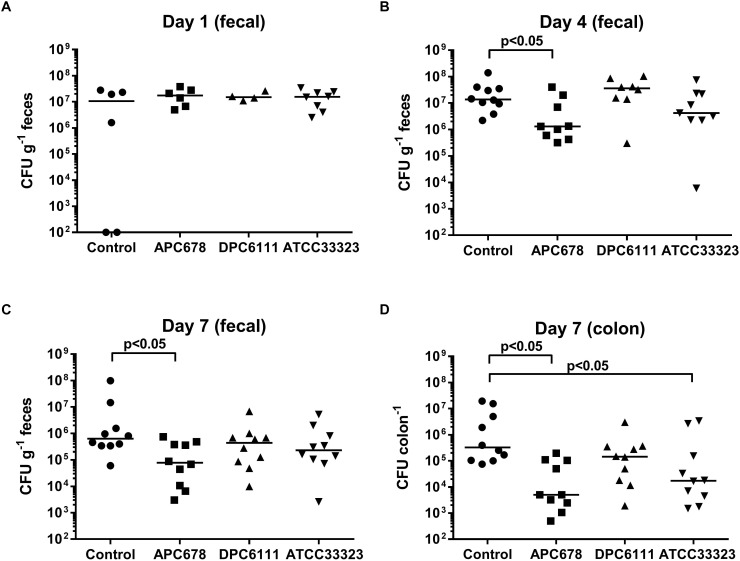
*Clostridium difficile* detected during fecal shedding. *C. difficile* detected in mouse feces (CFU g^-1^ feces) following **(A)** 24 h, **(B)** 4 days, and **(C)** 7 days administration of the test strains (or control); and **(D)** *C. difficile* levels in mouse colon (CFU colon^-1^) following 7 days administration of test strains (or control). Control: 10% RSM only; APC 678: *L. gasseri* APC 678; DPC 6111: *L. rhamnosus* DPC 6111 and ATCC 33323: *L. gasseri* ATCC 33323. Horizontal bar (^__^) represents the median.

The ability of *L. gasseri* APC 678 and *L. rhamnosus* DPC 6111 to reduce *C. difficile* shedding in a murine model was investigated. In addition, the well characterized strain *L. gasseri* ATCC 33323 (Azcarate-Peril et al., 2008), was selected as a control for the animal study. Levels of *C. difficile* shedding in the feces, total viable *C. difficile* in the mouse colon and changes in the microbiota composition of the mouse cecum were assessed. Mice were infected with ∼5 × 10^5^ CFU of *C. difficile* and at day 1 the mean *C. difficile* counts were ∼10^7^ CFU g^-1^ feces in all groups, which compares well with *C. difficile* counts in murine studies where the animals received clindamycin or metronidazole prior to infection with *C. difficile* (Schubert et al., 2015). There was no significant reduction in the median counts of *C. difficile* shed in the feces between the control and the *Lactobacillus*-fed mice after 24 h (Figure 2A). However, after 4 and 7 days, the presence of *L. gasseri* APC 678 significantly reduced *C. difficile* fecal shedding [11-fold (*p* = 0.022) and 8-fold (*p* = 0.017), respectively; fold reduction was calculated with median data] compared to the control mice, while there was no significant reduction in *C. difficile* in the feces of those mice receiving either *L. rhamnosus* DPC 6111 or *L. gasseri* ATCC 33323 compared to the control mice (Figure 2B,C). It was also noted that, by day 7, both *L. gasseri* APC 678 and *L. gasseri* ATCC 33323 significantly reduced the numbers of *C. difficile* that had adhered to the colon (*p* = 0.003 and *p* = 0.014, respectively; Figure 2D).

**Figure 3:**
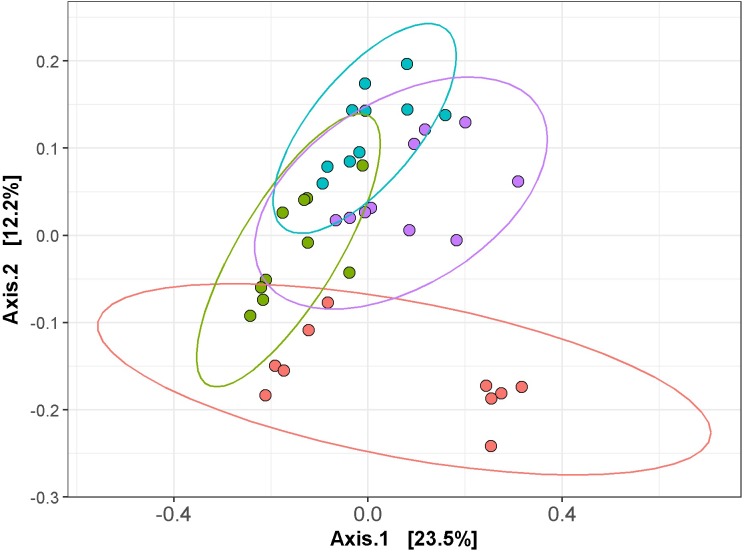
Principal coordinates analysis (PCoA) of the mouse cecal sequencing data at day 7, based on unweighted UniFrac distances, generated with phyloseq in R (version 3.2.2). The control and test groups are represented by color; control (red), *L. gasseri* APC 678 (green), *L. rhamnosus* DPC 6111 (blue) and *L. gasseri* ATCC 33323 (purple) and ggplot2 in R was used to compute the 95% confidence interval for the ellipses.

Following total metagenomic DNA extraction of the cecal contents, V3-V4 16S rRNA gene amplicons were generated and sequenced using the Illumina MiSeq. Diversity, richness and coverage estimations were calculated for each data set (Table 3), all of which indicated good sample richness throughout and the presence of a diverse microbiota. Interestingly, the Simpson and Shannon diversity metrics were significantly higher in the *L. gasseri* APC 678-fed mice compared to the control mice, and all alpha diversity indices tested were significantly increased in the mice fed *L. gasseri* APC 678 when compared to the those fed *L. gasseri* ATCC 33323 or *L. rhamnosus* DPC 6111, indicating that *L. gasseri* APC 678 had a greater positive impact on diversity than the other lactobacilli tested. Beta-diversity was estimated using distance matrices built from unweighted Unifrac distances and subsequently PCoA was performed on the distance matrices (Figure 3). Every effort was made to standardize the mice prior to (gender, source, age, antibiotic administration) and during the treatment and despite possible cage effect in the control, the groups clustered on the basis of the strain administered.

**Table 3:** Alpha diversity indices for sequencing coverage and microbiota diversity from cecum samples at Day 7 from control and test mice.

Alpha diversity index	Control	*L. gasseri* APC 678	*L. rhamnosus* DPC 6111	*L. gasseri* ATCC 33323
Chao1 richness estimate	210 ± 79	272 ± 40^a^	194 ± 38	168 ± 45
Simpson diversity index	0.92 ± 0.03	0.94 ± 0.05*,^a^	0.92 ± 0.04	0.89 ± 0.06
Shannon diversity index	4.77 ± 0.70	5.45 ± 0.72*,^a^	4.69 ± 0.52	4.41 ± 0.75
PD whole tree	11.69 ± 3.59	14.06 ± 1.71^a^	11.06 ± 1.63	9.73 ± 1.98
Number of observed species	197 ± 83	259 ± 39^a^	182 ± 37	153 ± 47

*Average and standard deviation of results for 10 mice per treatment are represented. ^∗^Significantly different compared to the control; ^*a*^significantly different compared to *L. rhamnosus* DPC 6111 and *L. gasseri* ATCC 33323; non-parametric Mann–Whitney test was used to estimate the relationship between the groups; statistical significance was accepted at *p* < 0.05*.

Sequence analysis revealed that the microbiota comprised of 7 main phyla (Table 4, Supplementary Figure 1, and Supplementary Table 3), with Firmicutes and Bacteroidetes dominating, and relative abundance corresponding to 28–55% and 43–71%, respectively. Unlike the group fed *L. gasseri* APC 678, where no significant change in the abundance of Firmicutes or Bacteroidetes relative to controls was observed, the groups fed *L. rhamnosus* DPC 6111 or *L. gasseri* ATCC 33323 showed a significant decrease in the relative abundance of Firmicutes (*p* = 0.002 and 0.019, respectively) and a significant increase in Bacteroidetes (*p* = 0.002 and 0.023, respectively) relative to the control. The relative abundance of the phylum Proteobacteria significantly decreased in the mice fed APC 678 or DPC 6111 relative to the control mice or the animals fed ATCC 33323, which showed an increase in Proteobacteria relative to the control. This pattern was mirrored in the significant reduction of the relative abundance of the genera *Escherichia/Shigella* in the groups fed *L. gasseri* APC 678 or *L. rhamnosus* DPC 6111. However, the decrease in the relative abundance of *Escherichia/Shigella* in the *L. gasseri* ATCC 33233-fed group was not significant. A diverse range of microbial families (Supplementary Figure 2 and Supplementary Table 4) and genera (Supplementary Figure 3 and Supplementary Table 5) were also detected across the four feeding groups. A number of statistical differences were found at family and genus OTU levels in the *Lactobacillus*-fed groups compared to the control (Table 4). The relative abundance of *Peptostreptococcaceae* was significantly reduced in all *Lactobacillus*-fed groups. This family encompasses *C. difficile*. The relative abundance of the genus *Alistipes* significantly increased in all *Lactobacillus*-fed groups relative to the control. The relative abundance of *Rikenellaceae* RC9 gut group also significantly increased in the *L. gasseri* APC 678 and *L. rhamnosus* DPC 6111 groups compared to the control, with the largest increase associated with the *L. gasseri* APC 678-fed group. The relative abundance of *Roseburia*, known to be associated with SCFA production (Rios-Covian et al., 2016), significantly increased in the *L. gasseri* APC 678-fed group only. In addition, the relative abundance of *Oscillibacter* significantly increased in the groups fed either *L. gasseri* APC 678 or *L. rhamnosus* DPC 6111 but not in the *L. gasseri* ATCC 33323-fed group (Table 4).

**Table 4:** Relative abundance (%) at bacterial phylum, family and genus level in the cecum at Day 7 of the control and test mice (*Lactobacillus gasseri* APC 678, *Lactobacillus rhamnosus* DPC 6111 and *Lactobacillus gasseri* ATCC 33323).

Group	Control	APC 678	DPC 6111	ATCC 33323
	
	relative abundance (%)			
**Phylum:**				
Firmicutes	54.51	37.22	28.44*	31.29*
Bacteroidetes	43.17	62.24	70.62*	66.25*
Proteobacteria	0.47	0.19*	0.09*	1.29
Actinobacteria	0.26	0.18	0.19	0.13*


**Family:**				


*Lachnospiraceae*	40.35	27.46	20.14*	23.60*
S24-7	24.10	31.18	40.72*	23.21
*Bacteroidaceae*	9.29	21.60	18.61	28.21*
Uncultured *Clostridiales*	2.01	0.64	0.21*	0.58*
*Rikenellaceae*	1.86	7.31*	6.52*	8.11*
*Erysipelotrichaceae*	1.80	0.62*	0.83*	0.95*
*Peptostreptococcaceae*	0.60	0.08*	0.02*	0.05*
*Alcaligenaceae*	0.29	0.09*	0.069*	1.24
*Enterobacteriaceae*	0.18	0.01*	0.02*	0.06
*Bifidobacteriaceae*	0.13	0.04*	0.02*	0.07*
*Enterococcaceae*	0.03	0.02	0.01*	0.01
*Peptococcaceae*	0.00	0.04*	0.02	0.07
*Prevotellaceae*	0.00	0.53	0.56*	0.00
*Xanthomonadaceae*	0.00	0.06*	0.00	0.00


**Genus:**				
Uncultured *Lachnospiraceae*	29.99	20.93	14.78*	18.00*
Uncultured S24-7	24.10	31.18	40.72*	23.21
*Bacteroides*	9.29	21.60	18.61	28.21*
*Ruminococcaceae Incertae Sedis*	5.36	2.19*	1.44*	2.01*
Uncultured *Clostridiales*	2.01	0.64	0.21*	0.58*
*Alistipes*	1.41	3.40*	4.95*	6.03*
Uncultured *Ruminococcaceae*	1.08	2.03*	0.77	1.31
*Peptostreptococcaceae Incertae Sedis*	0.60	0.08*	0.02*	0.05*
*Anaerotruncus*	0.60	1.09	1.85*	0.76
*Rikenellaceae* RC9 gut group	0.45	3.90*	1.57*	2.08
*Oscillibacter*	0.36	1.15*	1.81*	0.79
*Parasutterella*	0.29	0.09*	0.07*	1.24
*Flavonifractor*	0.25	0.01*	0.01*	0.09
*Roseburia*	0.21	0.90*	0.21	0.29
*Escherichia-Shigella*	0.18	0.01*	0.02*	0.06
uncultured *Erysipelotrichaceae*	0.14	0.04*	0.05*	0.04*
*Bifidobacterium*	0.13	0.04*	0.02*	0.07*
*Enterococcus*	0.03	0.02	0.007*	0.01
uncultured *Peptococcaceae*	0.00	0.03*	0.01	0.07
*Prevotella*	0.00	0.53	0.56*	0.00
*Hydrogenoanaerobacterium*	0.00	0.004*	0.00	0.00
*Stenotrophomonas*	0.00	0.06*	0.00	0.00

*Only phyla, families and genera with significant differences compared to the control mice are represented. ^∗^Significantly different compared to the control; significance was determined by *p* < 0.05*.

## Discussion

The increasing frequency of CDI in hospitals, facilities for the elderly and more recently community settings (Chitnis et al., 2013), combined with the resulting health and economic burdens, warrants the search for alternative treatments for CDI. The use of microbial therapy as a preventive measure to mitigate the increasing prevalence of CDI is one such avenue under investigation. Surveys of the literature have shown that for pediatric use, *Lactobacillus* strains significantly prevented antibiotic-associated diarrhea and CDI (Goldenberg et al., 2013). However, while there was some evidence that microbial therapy was effective in preventing primary CDI in adults, there was insufficient evidence to confirm the efficacy of these strains to prevent recurrent CDI (Evans and Johnson, 2015; Goldstein et al., 2015).

The concept of strain-specific benefits is not new and has already been shown to be true of live therapeutics for other applications (Wall et al., 2012). In this context, our study pre-screened a range of *Lactobacillus* species for their ability to inhibit *C. difficile* with a view to identifying potential strains to target CDI in humans. To that end, we developed a medium which allowed the survival of both *Lactobacillus* and *C. difficile* strains in co-culture, without the decrease in pH normally associated with the growth of lactobacilli in other media, such as MRS or RCM. We identified 4 strains from 60 *Lactobacillus* strains screened (2 *L. gasseri* strains, 1 *L. rhamnosus* strain, and 1 *L. paracasei* strain), all of human origin, which negatively impacted the growth of *C. difficile in vitro.* Among the strains screened was *L. gasseri* ATCC 33323, which has a number of traits encoded on its genome which are important for its survival and retention in the GIT (Azcarate-Peril et al., 2008). Interestingly, while seven strains of *L. gasseri* were screened *in vitro* only two *L. gasseri* strains inhibited *C. difficile*, lending credence to the theory that not all strains of the same species have the same effect (Arnold et al., 2018; Liu et al., 2018).

Two lead candidates from the *in vitro* work, namely *L. gasseri* APC 678 and *L. rhamnosus* DPC 6111, were selected for *in vivo* analysis. These strains have the added advantage of surviving in higher numbers during simulated gastric transit, at low pH and in the presence of bile and the digestive enzymes encountered in the stomach and upper GIT. The increased survival rate in these environments in the presence of milk is a further bonus as fermented milk products such as yogurts and cheese are often used as vehicles for oral delivery of live bacteria (Gardiner et al., 1998; Hickson et al., 2007). The health impacts of these *Lactobacillus* species have been previously investigated (Bravo et al., 2011; Kadooka et al., 2013; Kechagia et al., 2013; Kobatake et al., 2017; Wang et al., 2017).

As a first step to determining the efficacy of these strains with respect to decreasing CDI *in vivo*, the strains (*L. gasseri* APC 678, *L. rhamnosus* DPC 6111 and the aforementioned well-characterized *L. gasseri* ATCC 33323) were tested for their ability to reduce fecal shedding of *C. difficile* in a murine model of CDI over 7 days. During this period, *C. difficile* VPI 10463 did not result in the manifestation of obvious signs of disease in the mice, and as such was regarded for this purpose as a colonization model of CDI. *Clostridium difficile* levels were monitored in the feces by culturing at days 1, 4 and 7 and in the mouse colon by culturing at Day 7. Sequence analysis was carried out on the mouse cecal contents at Day 7. The ability to reduce fecal shedding of *C. difficile* was significant, when compared to the control group fed RSM, in those animals fed *L. gasseri* APC 678 4 days post-infection. This reduction was maintained for up to 7 days, at which time the animals were euthanized. No significant effect was seen in terms of fecal shedding of *C. difficile* in those mice fed either *L. gasseri* 33323 or *L. rhamnosus* DPC 6111. In the initial *in vitro* co-culture assays, *L. rhamnosus* DPC 6111 had an equal inhibitory effect to *L. gasseri* APC 678. However, this was not reflected *in vivo* as the *L. gasseri* APC 678 out-performed the *L. rhamnosus* strain. Interestingly, when the level of viable *C. difficile* in the colon was assessed the numbers were significantly reduced in those mice that were fed either of the *L. gasseri* strains, which may be as a result of competitive exclusion of *C. difficile* by the *L. gasseri* strains. The absence of evidence of bacteriocin activity *in vitro* would suggest that inhibition of *C. difficile* by bacteriocins is not responsible. Other possible means by which the *L. gasseri* protect the host against *C. difficile* include stimulation of the host’s immune response, competition for nutrients and protection of the integrity of the gastrointestinal mucosa.

CDI is normally the result of changes to the gut microbiota as a result of broad-spectrum antibiotic treatment which results in a decrease in microbial diversity (Rea et al., 2012). One desired function of a live therapeutic in a disease state would be to increase microbial diversity, thereby reducing the ability of *C. difficile* to survive and multiply due to competition for nutrients. Compositional sequencing showed that in the control and the test mice there was a diverse microbiota despite the prior administration of antibiotics to make the animals more susceptible to infection. Also, while every effort was made to standardize the mice prior to and during the study; the PCoA plot of the cecal sequencing data for the control mice at Day 7 did suggest a separation based on housing. Despite this the sequencing data from the groups were seen to be statistically different based on the strain administered. *L. gasseri* APC 678 increased diversity for all the indices tested, including the number of observed species, compared to the other strains studied. It has been recognized that a decrease in diversity has been linked to CDI (Rea et al., 2012; Gu et al., 2016). However, unlike the mice fed *L. gasseri* APC 678, where no significant change in the relative abundance of the main phyla was observed when compared to the control, there was a significant change in the Firmicutes and Bacteroides levels in the groups fed either *L. rhamnosus* DPC 6111 or *L. gasseri* ATCC 33323 when compared to the control with a significant increase in Bacteroidetes. The importance, if any, of this shift is unclear. However, an increase in the abundance of Proteobacteria (and the genera *Escherichia/Shigella)* has been associated with antibiotic use (Cotter et al., 2012) and the animals fed either *L. gasseri* APC 678 or *L. rhamnosus* DPC 6111 showed a significant decrease in the relative abundance of Proteobacteria and *Escherichia/Shigella*, while the change in the animals fed *L. gasseri* ATCC 33323 was not significant, establishing that strains of the same species exert differing effects in this regard. A study by Schubert and colleagues observed that when the gut microbiota in a murine model was altered as a result of antibiotic administration, populations of *Porphyromonadaceae*, *Lachnospiraceae*, *Lactobacillus* and *Alistipes* protected against *C. difficile* colonization (Schubert et al., 2015). While our study showed that *Lachnospiraceae* were significantly reduced in the groups fed *L. rhamnosus* DPC 6111 and *L. gasseri* ATCC 33323 but not the *L. gasseri* APC 678-fed group, it was notable that the relative abundance of *Alistipes* was significantly increased in all *Lactobacillus*-fed animals relative to the control group. In a study in which the microbiota profile of CDI patients were compared, the relative abundance of *Alistipes* significantly decreased in the CDI patients (*n* = 25) compared to non-CDI patients (*n* = 30) not in receipt of antibiotics (Schubert et al., 2015; Milani et al., 2016). The increase in the relative abundance of genera involved in the production of SCFAs, such as *Roseburia* (in *L. gasseri* APC 678-fed group) and *Oscillibacter* (*L. gasseri* APC 678 or *L. rhamnosus* DPC 6111-fed groups) would suggest that lactobacilli can exert beneficial effects which are strain rather than species specific.

In the fight against CDI it is likely that live therapeutics, either as well characterized single/multiple strains as described here or the more complex, less defined microbiota in FMT will play a role in addressing the reduction in microbial diversity in the GIT that results from broad spectrum antibiotic treatment leading to CDI. The interactions between the gut microbiome and the host are complex and FMT may therefore have unintended consequences in a patient after successful FMT due to alteration of the gut microbiota. FMT in experimental animals has shown that immunologic, behavioral and metabolic phenotypes can be transferred from donor to recipient, which may not always be beneficial to the recipient in the long-term (Collins et al., 2013; Pamer, 2014; Di Luccia et al., 2015). Therefore, there are advantages to using well characterized strains with QPS status (EFSA, 2007) with proven efficacy against *C. difficile in vivo* which translate into positive changes in the gut microbiota profile.

## Author Contributions

DA, MR, EM, BK, PDC, CH and RR conceived and designed the study. DA, MR, LQ, MC and PGC conducted the experiments and interpreted and analyzed the data. ÓO’S carried out bioinformatic analysis. LQ, MC, DA and MR wrote the manuscript. All authors read, corrected, and approved the final manuscript.

## Conflict of Interest Statement

SFI had no role in the study design, data collection and analysis, decision to publish, or preparation of the manuscript. This work was part-funded by a research grant from Alimentary Health Ltd, of which EM and BK are employees. Both EM and BK contributed to the preparation of the manuscript. Five co-authors; RR, MR, CH, EM and DA are named on a patent application relating to this work (patent application number: WO/2018/115361). The remaining authors declare that the research was conducted in the absence of any commercial or financial relationships that could be construed as a potential conflict of interest.

## Abbreviations

BHI: brain heart infusion
CCEY: cefoxitin cycloserine and egg yolk agar
CDI: *C. difficile* infection
CLA: conjugated linoleic acid
EFSA: European Food Safety Authority
ENA: European Nucleotide Archive
ESCMID: European Society of Clinical Microbiology and Infectious Diseases
FMT: fecal microbiota transplantation
GABA: gamma-aminobutyric acid
GIT: gastrointestinal tract
HPRA: Health Products Regulatory Authority of Ireland
MGM: minimal growth medium
MRD: maximum recovery diluent
MRS: de Man Rogosa Sharpe
OD: optical density
OTU: operational taxonomical unit
PBS: phosphate buffered saline
PCoA: principal coordinates analysis
QPS: qualified presumption of safety
RCM: reinforced clostridial medium
RSM: reconstituted skim milk
SCFA: short chain fatty acid
SFI: Science Foundation Ireland.
