# Supplementary material for: Lactobacillus gasseri APC 678 Reduces Shedding of the Pathogen Clostridium difficile in a Murine Model
Source: Front Microbiol. 2019 Feb 20;10:273. doi: 10.3389/fmicb.2019.00273 (PMC6391587; doi:10.3389/fmicb.2019.00273)
Supplement: Supplementary file 1 [file Data_Sheet_1.pdf]

Supplementary Material

Lactobacillus gasseri APC 678 Reduces Shedding of the Pathogen *Clostridium difficile* in a Murine Model

Lisa Quigley[#], Mairéad Coakley[#], Debebe Alemayehu, Mary C. Rea, Patrick G. Casey, Órla O'Sullivan, Eileen Murphy, Barry Kiely, Paul D. Cotter, Colin Hill and R. Paul Ross*

***Correspondence:**
Prof. R. Paul Ross
p.ross@ucc.ie

[#] LQ and MC are joint first authors

1.1 Supplementary Figures

Supplementary Figure 1: Relative abundance (%) of bacterial phyla in the cecum at Day 7 of the control and test mice (*Lactobacillus gasseri* APC 678, *Lactobacillus rhamnosus* DPC 6111 and *Lactobacillus gasseri* ATCC 33323).

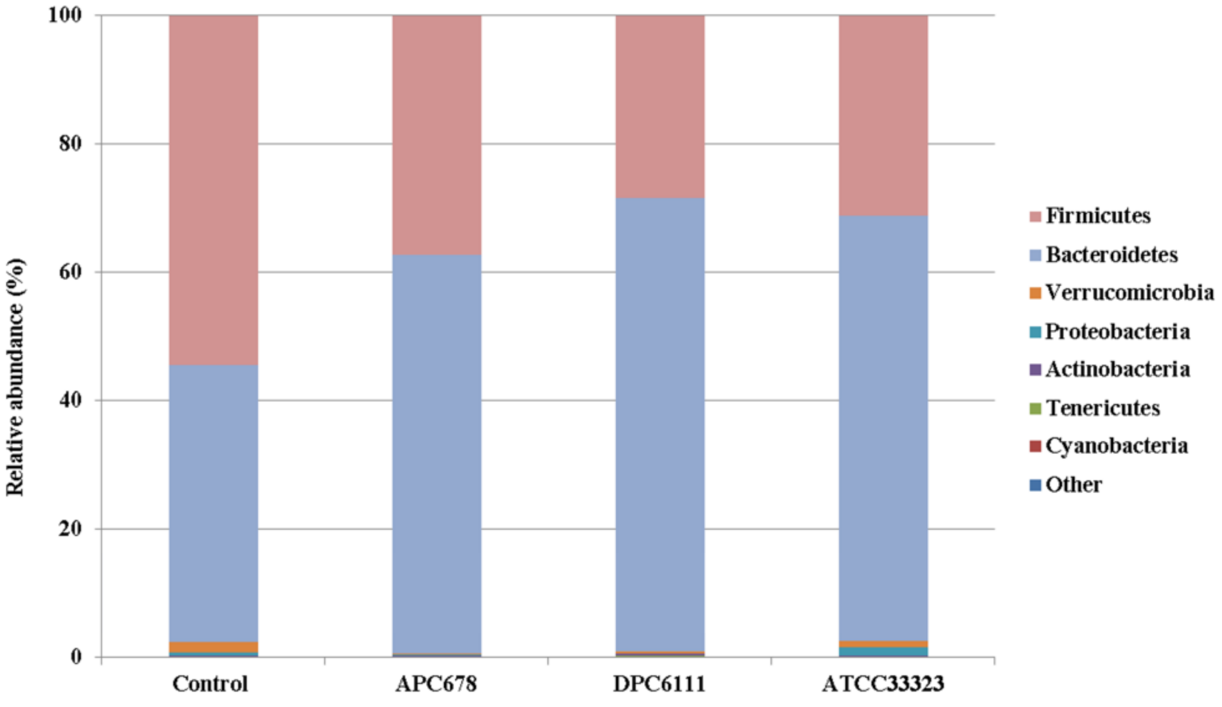

Supplementary Figure 2: Relative abundance (%) of bacterial families in the cecum at Day 7 of the control mice and test mice (*Lactobacillus gasseri* APC 678, *Lactobacillus rhamnosus* DPC 6111 and *Lactobacillus gasseri* ATCC 33323).

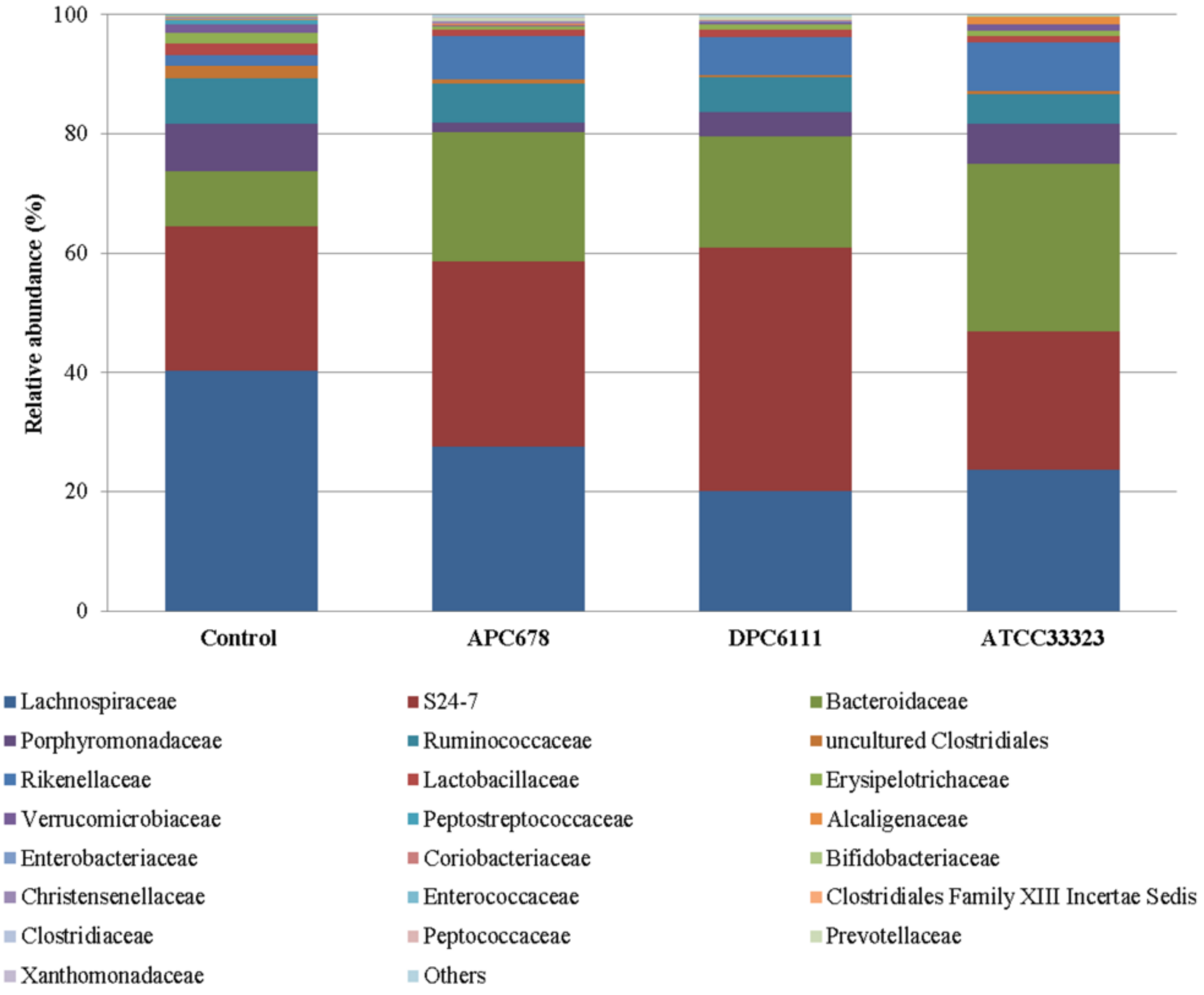

Supplementary Figure 3: Relative abundance (%) of bacterial genera in the cecum at Day 7 of the control mice and test mice (*Lactobacillus gasseri* APC 678, *Lactobacillus rhamnosus* DPC 6111 and *Lactobacillus gasseri* ATCC 33323).

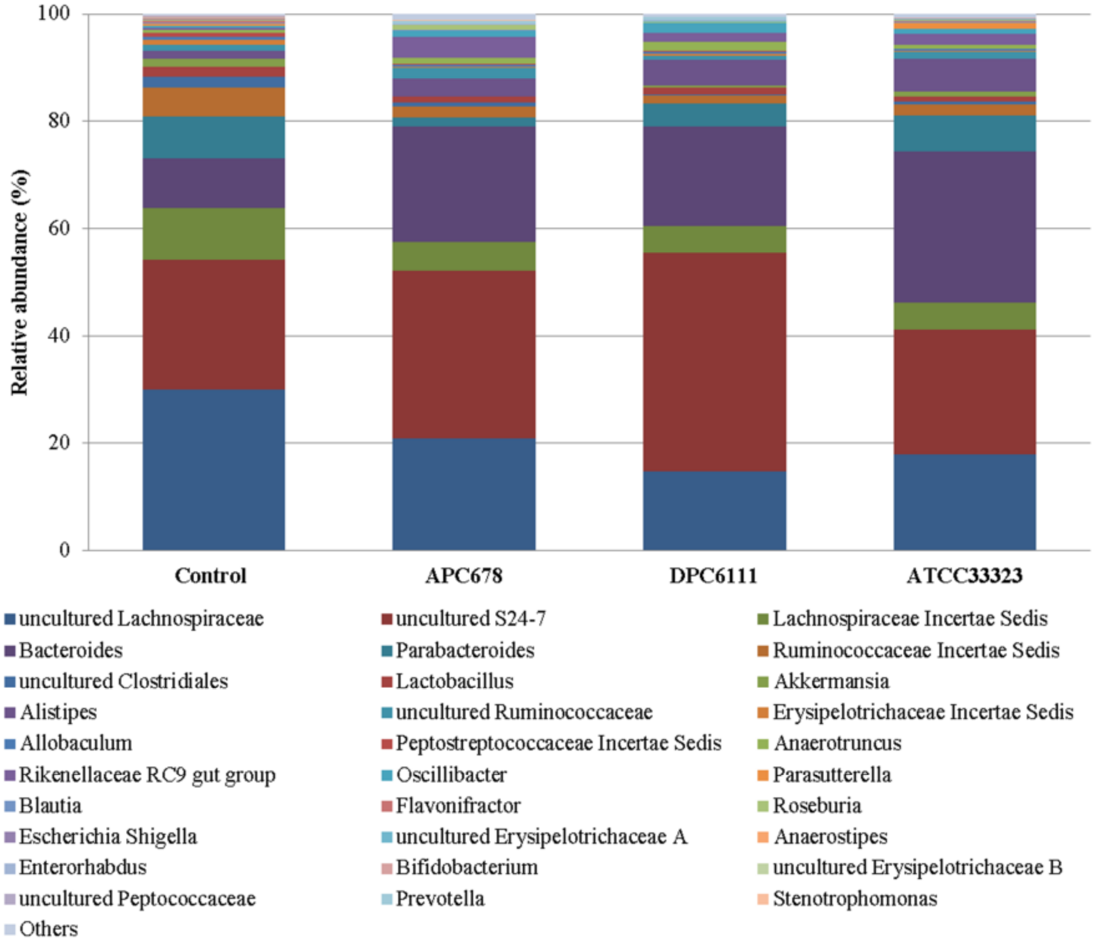

1.2 Supplementary Tables

Supplementary Table 1: Source and origin of bacterial strains used during this study.

**Bacterial strain**	**Source**	**Origin**
Clostridium difficile EM304 (ribotype 027)	ELDERMET Culture Collection[1]	elderly adult feces
Clostridium difficile VPI 10463 (ATCC® 43255-FZ™)	American Type Culture Collection[2]	human abdominal wound
L. gasseri APC 678	APC Culture Collection[1]	human feces
L. paracasei APC 1483	APC Culture Collection	human feces
L. rhamnosus DPC 6111	DPC Culture Collection[1]	human feces
L. gasseri DPC 6112	DPC Culture Collection	human feces
L. gasseri ATCC 33323	American Type Culture Collection	human gastrointestinal tract

[1]housed at Teagasc, Moorepark, Fermoy, Co. Cork, Ireland; [2]Manassas, VA 20110, USA.

Supplementary Table 2: Target strains, growth medium and incubation conditions for well diffusion assays to detect bacteriocin production by *L. gasseri* APC 678 *in vitro*.

Target strain	Culture Collection code	Incubation conditions	Growth medium
Enterococcus faecium	LMG[1] 11423	37°C, anaerobic	MRS
Enterococcus mundtii	LMG 10758	37°C, anaerobic	MRS
Enterococcus saccharolyticus	LMG 11427	37°C, anaerobic	MRS
Lactobacillus acidophilus	LMG 9433	37°C, anaerobic	MRS
Lactobacillus agilis	LMG 9186	37°C, anaerobic	MRS
Lactobacillus amylovorus	LMG 9496	37°C, anaerobic	MRS
Lactobacillus casei	LMG 6904	37°C, anaerobic	MRS
Lactobacillus crispatus	LMG 9479	37°C, anaerobic	MRS
Lactobacillus delbrueckii subsp. *bulgaricus*	LMG 6901	37°C, anaerobic	MRS
Lactobacillus delbrueckii subsp. *lactis*	LMG 7942	37°C, anaerobic	MRS
Lactobacillus fermentum	LMG 6902	37°C, anaerobic	MRS
Lactobacillus plantarum	LMG 6907	37°C, anaerobic	MRS
Lactobacillus rhamnosus	ATCC[2] 53103	37°C, anaerobic	MRS
Listeria innocua	DPC[3] 3572	37°C, aerobic	BHI*
Micrococcus luteus	DPC 6275	30°C, aerobic	BHI
Salmonella typhimurium	DPC 6048	37°C, aerobic	BHI
Staphylococcus aureus	DPC 5246	37°C, aerobic	BHI

[1]LMG Culture Collection, B-9000 Gent, Belgium; [2]American Type Culture Collection, Manassas, VA 20110, USA and [3]DPC Culture Collection, Teagasc, Moorepark, Fermoy, Co. Cork, Ireland. *BHI: Brain heart infusion medium, Merck, Darmstadt, Germany.

Supplementary Table 3: Relative abundance (%) of bacterial phyla in the cecum at Day 7 of the control and test mice (*Lactobacillus gasseri* APC 678, *Lactobacillus rhamnosus* DPC 6111 and *Lactobacillus gasseri* ATCC 33323). Average and standard error of the mean (SEM) of results for 10 mice per treatment are represented.

**Group**	**Control**	**APC 678**	**DPC 6111**	**ATCC 33323**
	**relative abundance (%)**			
**Firmicutes**	54.51±7.32	37.22±4.18	28.44±2.32*	31.29±3.63*
**Bacteroidetes**	43.17±7.19	62.24±4.19	70.62±2.31*	66.25±3.88*
**Verrucomicrobia**	1.53±1.42	0.08±0.03	0.35±0.12	0.93±0.76
**Proteobacteria**	0.47±0.08	0.19±0.06*	0.09±0.03*	1.29±0.44
**Actinobacteria**	0.26±0.05	0.18±0.03	0.19±0.04	0.13±0.03*
**Tenericutes**	0.01±0.01	0.03±0.02	0.26±0.14	0.10±0.07
**Cyanobacteria**	0.00±0.00	0.00±0.00	0.00±0.00	0.00±0.00
**Other**	0.04±0.01	0.05±0.01	0.05±0.01	0.02±0.01

*significantly different compared to the control; significance was determined by $p<0.05$.

Supplementary Table 4: Relative abundance (%) of bacterial families in the cecum at Day 7 of the control and test mice (*Lactobacillus gasseri* APC 678, *Lactobacillus rhamnosus* DPC 6111 and *Lactobacillus gasseri* ATCC 33323). Average and standard error of the mean (SEM) of results for 10 mice per treatment are represented.

**Group**	**Control**	**APC 678**	**DPC 6111**	**ATCC 33323**
	**relative abundance (%)**			
Lachnospiraceae	40.346±5.285	27.461±3.459	20.141±2.049*	23.600±3.273*
**S24-7**	24.104±5.359	31.182±2.779	40.720±3.787*	23.214±4.942
Bacteroidaceae	9.289±3.278	21.603±4.812	18.614±3.699	28.214±5.646*
Porphyromonadaceae	7.918±2.407	1.611±0.396	4.213±1.364	6.710±1.656
Ruminococcaceae	7.743±1.458	6.576±1.054	5.874±0.974	4.957±0.678
**uncultured *Clostridiales***	2.008±0.655	0.639±0.130	0.213±0.082*	0.582±0.284*
Rikenellaceae	1.859±0.696	7.314±0.900*	6.515±0.753*	8.112±1.439*
Lactobacillaceae	1.835±0.515	1.078±0.480	1.259±0.361	1.036±0.229
Erysipelotrichaceae	1.804±0.370	0.616±0.110*	0.829±0.188*	0.948±0.341*
Verrucomicrobiaceae	1.531±1.419	0.084±0.029	0.354±0.121	0.928±0.755
Peptostreptococcaceae	0.603±0.245	0.078±0.054*	0.019±0.013*	0.050±0.031*
Alcaligenaceae	0.286±0.060	0.085±0.043*	0.069±0.032*	1.235±0.429
Enterobacteriaceae	0.177±0.072	0.010±0.004*	0.017±0.009*	0.055±0.026
Coriobacteriaceae	0.130±0.040	0.140±0.025	0.167±0.036	0.063±0.018
Bifidobacteriaceae	0.126±0.031	0.039±0.016*	0.024±0.011*	0.065±0.041*
Christensenellaceae	0.103±0.046	0.375±0.178	0.000±0.000	0.012±0.006
Enterococcaceae	0.030±0.008	0.023±0.009	0.007±0.003*	0.013±0.007
Clostridiales* Family XIII *Incertae Sedis	0.019±0.006	0.028±0.005	0.029±0.010	0.019±0.005
Clostridiaceae	0.016±0.010	0.000±0.000	0.002±0.002	0.001±0.001
Anaeroplasmataceae	0.013±0.011	0.033±0.023	0.258±0.140	0.097±0.075
Oxalobacteraceae	0.005±0.002	0.000±0.000	0.000±0.000	0.000±0.000
Peptococcaceae	0.004±0.002	0.041±0.009*	0.020±0.007	0.066±0.049
Rhodospirillaceae	0.002±0.001	0.008±0.004	0.000±0.000	0.000±0.000
Staphylococcaceae	0.002±0.001	0.288±0.149	0.045±0.035	0.000±0.000
Streptococcaceae	0.001±0.001	0.001±0.000	0.000±0.000	0.000±0.000
***Rickettsiales* mitochondria**	0.001±0.001	0.000±0.000	0.000±0.000	0.000±0.000
**Cyanobacteria 4C0d-2 uncultured**	0.001±0.001	0.000±0.000	0.000±0.000	0.000±0.000
Moraxellaceae	0.001±0.001	0.005±0.002	0.000±0.000	0.000±0.000
Planococcaceae	0.000±0.000	0.012±0.005	0.000±0.000	0.000±0.000
Prevotellaceae	0.000±0.000	0.533±0.198	0.561±0.201*	0.001±0.001
***Bacteroidales* ratAN060301C**	0.000±0.000	0.000±0.000	0.000±0.000	0.000±0.000
Bacillaceae	0.000±0.000	0.001±0.001	0.000±0.000	0.000±0.000
Methylobacteriaceae	0.000±0.000	0.000±0.000	0.000±0.000	0.001±0.001
Comamonadaceae	0.000±0.000	0.002±0.001	0.000±0.000	0.000±0.000
Desulfovibrionaceae	0.000±0.000	0.024±0.024	0.000±0.000	0.000±0.000
Xanthomonadaceae	0.000±0.000	0.057±0.048*	0.001±0.001	0.000±0.000
**Others**	0.045±0.014	0.051±0.005	0.047±0.010	0.018±0.010

*significantly different compared to the control; significance was determined by p<0.05.

Supplementary Table 5: Relative abundance (%) of bacterial genera in the cecum at Day 7 of the control and test mice (*Lactobacillus gasseri* APC 678, *Lactobacillus rhamnosus* DPC 6111 and *Lactobacillus gasseri* ATCC 33323). Average and standard error of the mean (SEM) of results for 10 mice per treatment are represented.

**Genus**	**Control**	**APC 678**	**DPC 6111**	**ATCC 33323**
	**relative abundance (%)**			
**uncultured *Lachnospiraceae* A**	29.985±3.699	20.933±3.149	14.777±2.014*	18.000±3.312*
**uncultured S24-7**	24.104±5.359	31.182±2.779	40.720±3.787*	23.214±4.942
Lachnospiraceae Incertae Sedis	9.665±2.069	5.321±0.918	4.994±1.722	4.906±0.772
Bacteroides	9.289±3.278	21.603±4.812	18.614±3.699	28.214±5.646*
Parabacteroides	7.917±2.407	1.611±0.396	4.213±1.364	6.710±1.656
Ruminococcaceae Incertae Sedis	5.364±1.393	2.185±0.485*	1.441±0.300*	2.014±0.454*
**uncultured *Clostridiales***	2.008±0.655	0.639±0.130	0.213±0.082*	0.582±0.284*
Lactobacillus	1.835±0.515	1.078±0.480	1.259±0.361	1.036±0.229
Akkermansia	1.531±1.419	0.084±0.029	0.354±0.121	0.928±0.755
Alistipes	1.406±0.507	3.395±0.343*	4.949±0.585*	6.034±0.907*
**uncultured *Ruminococcaceae***	1.081±0.189	2.031±0.339*	0.771±0.286	1.308±0.441
Erysipelotrichaceae Incertae Sedis	0.953±0.392	0.171±0.062	0.290±0.060	0.268±0.087
Allobaculum	0.691±0.346	0.401±0.133	0.449±0.150	0.294±0.139
Peptostreptococcaceae Incertae Sedis	0.603±0.245	0.078±0.054*	0.019±0.013*	0.050±0.031*
Anaerotruncus	0.601±0.155	1.090±0.252	1.846±0.399*	0.755±0.154
***Rikenellaceae* RC9 gut group**	0.453±0.319	3.895±0.815*	1.566±0.590*	2.078±0.956
Oscillibacter	0.363±0.199	1.151±0.258*	1.805±0.421*	0.787±0.291
Parasutterella	0.286±0.060	0.085±0.043*	0.069±0.032*	1.235±0.429
Blautia	0.267±0.105	0.076±0.072	0.030±0.017	0.132±0.103
Flavonifractor	0.251±0.176	0.012±0.010*	0.007±0.005*	0.087±0.058
Roseburia	0.207±0.083	0.903±0.291*	0.212±0.104	0.288±0.228
Escherichia/Shigella	0.177±0.072	0.010±0.004*	0.017±0.009*	0.055±0.026
**uncultured *Erysipelotrichaceae* A**	0.138±0.030	0.036±0.008*	0.054±0.018*	0.039±0.009*
Anaerostipes	0.129±0.040	0.064±0.049	0.064±0.023	0.065±0.040
Enterorhabdus	0.128±0.040	0.135±0.023	0.159±0.034	0.059±0.017
Bifidobacterium	0.126±0.031	0.039±0.016*	0.024±0.011*	0.065±0.041*
**uncultured *Christensenellaceae***	0.102±0.046	0.375±0.178	0.000±0.000	0.008±0.005
Ruminococcus	0.082±0.045	0.102±0.072	0.001±0.001	0.005±0.004
Coprococcus	0.053±0.030	0.063±0.015	0.017±0.014	0.020±0.009
Marvinbryantia	0.038±0.007	0.099±0.042	0.047±0.031	0.188±0.079
Enterococcus	0.030±0.008	0.023±0.009	0.007±0.003*	0.013±0.007
Clostridiales* Family XIII *Incertae Sedis	0.019±0.006	0.025±0.004	0.029±0.010	0.012±0.004
**uncultured *Erysipelotrichaceae* B**	0.017±0.007	0.009±0.004	0.026±0.012	0.011±0.008
Clostridium	0.016±0.010	0.000±0.000	0.002±0.002	0.001±0.001
Anaeroplasma	0.013±0.011	0.033±0.023	0.258±0.140	0.097±0.075
Oxalobacter	0.005±0.002	0.000±0.000	0.000±0.000	0.000±0.000
Coprobacillus	0.004±0.004	0.000±0.000	0.010±0.010	0.337±0.275
**uncultured *Peptococcaceae***	0.004±0.002	0.025±0.005*	0.006±0.002	0.066±0.049

Acetitomaculum	0.002±0.001	0.000±0.000	0.000±0.000	0.000±0.000
Thalassospira	0.002±0.001	0.008±0.004	0.000±0.000	0.000±0.000
Staphylococcus	0.002±0.001	0.287±0.149	0.045±0.035	0.000±0.000
Streptococcus	0.001±0.001	0.001±0.000	0.000±0.000	0.000±0.000
Christensenella	0.001±0.001	0.000±0.000	0.000±0.000	0.004±0.002
**uncultured *Coriobacteriaceae***	0.001±0.001	0.005±0.002	0.007±0.005	0.004±0.003
Idiospermum	0.001±0.001	0.000±0.000	0.000±0.000	0.000±0.000
**uncultured Cyanobacteria 4C0d-2**	0.001±0.001	0.000±0.000	0.000±0.000	0.000±0.000
Acinetobacter	0.001±0.001	0.005±0.002	0.000±0.000	0.000±0.000
Sporosarcina	0.000±0.000	0.012±0.005	0.000±0.000	0.000±0.000
Barnesiella	0.000±0.000	0.000±0.000	0.000±0.000	0.000±0.000
Prevotella	0.000±0.000	0.533±0.198	0.561±0.201*	0.000±0.000
**uncultured *Prevotellaceae***	0.000±0.000	0.000±0.000	0.000±0.000	0.001±0.001
Rikenella	0.000±0.000	0.025±0.021	0.000±0.000	0.000±0.000
**uncultured *Bacteroidales* ratAN060301C**	0.000±0.000	0.000±0.000	0.000±0.000	0.000±0.000
Bacillus	0.000±0.000	0.001±0.001	0.000±0.000	0.000±0.000
Salinicoccus	0.000±0.000	0.001±0.001	0.000±0.000	0.000±0.000
Anaerovorax	0.000±0.000	0.000±0.000	0.000±0.000	0.001±0.001
**uncultured *Clostridiales* Family XIII *Incertae Sedis***	0.000±0.000	0.003±0.002	0.000±0.000	0.007±0.003
**uncultured *Lachnospiraceae* B**	0.000±0.000	0.002±0.002	0.000±0.000	0.000±0.000
Peptococcus	0.000±0.000	0.016±0.009	0.015±0.006	0.000±0.000
Hydrogenoanaerobacterium	0.000±0.000	0.004±0.002*	0.000±0.000	0.000±0.000
Subdoligranulum	0.000±0.000	0.000±0.000	0.003±0.002	0.000±0.000
Methylobacterium	0.000±0.000	0.000±0.000	0.000±0.000	0.001±0.001
Variovorax	0.000±0.000	0.002±0.001	0.000±0.000	0.000±0.000
Bilophila	0.000±0.000	0.024±0.024	0.000±0.000	0.000±0.000
Stenotrophomonas	0.000±0.000	0.057±0.048*	0.001±0.001	0.000±0.000
**Other**	0.045±0.014	0.051±0.005	0.047±0.010	0.018±0.010

*significantly different compared to the control; significance was determined by $p<0.05$.
